# From Benign Inflammatory Dermatosis to Cutaneous Lymphoma. DNA Copy Number Imbalances in Mycosis Fungoides versus Large Plaque Parapsoriasis

**DOI:** 10.3390/medicina57050502

**Published:** 2021-05-15

**Authors:** Georgiana Gug, Caius Solovan

**Affiliations:** 1 Research Center for the Morphologic Study of the Skin, Department of Dermatology and Venereology, “Victor Babeș” University of Medicine and Pharmacy, Eftimie Murgu Sq. No. 2, 300041 Timișoara, Romania; 2 Emergency City Hospital, University Clinic of Dermatology and Venereology, 300558 Timișoara, Romania

**Keywords:** CTCL, Mycosis fungoides, large plaque parapsoriasis, genes, copy number alterations, deletions, duplications, T-cell receptor

## Abstract

*Background and Objectives:* Mycosis fungoides (MF) and large plaque parapsoriasis (LPP) evolution provide intriguing data and are the cause of numerous debates. The diagnosis of MF and LPP is associated with confusion and imprecise definition. Copy number alterations (CNAs) may play an essential role in the genesis of cancer out of genes expression dysregulation. Objectives: Due to the heterogeneity of MF and LPP and the scarcity of the cases, there are an exceedingly small number of studies that have identified molecular changes in these pathologies. We aim to identify and compare DNA copy number alterations and gene expression changes between MF and LPP to highlight the similarities and the differences between these pathologies. *Materials and Methods:* The patients were prospectively selected from University Clinic of Dermatology and Venereology Timișoara, Romania. From fresh frozen skin biopsies, we extracted DNA using single nucleotide polymorphism (SNP) data. The use of SNP array for copy number profiling is a promising approach for genome-wide analysis. *Results:* After reviewing each group, we observed that the histograms generated for chromosome 1–22 were remarkably similar and had a lot of CNAs in common, but also significant differences were seen. *Conclusions:* This study took a step forward in finding out the differences and similarities between MF and LPP, for a more specific and implicitly correct approach of the case. The similarity between these two pathologies in terms of CNAs is striking, emphasizing once again the difficulty of approaching and differentiating them.

## 1. Introduction

Primary cutaneous lymphoma (PCL) is a subtype of non-Hodgkin lymphoma and represents progressive clonal proliferation in the skin, of B-cells, T-cells or NK-cells. Morphologically, the cutaneous tissue can be the initial and unique organ affected or the secondary one, after a previous organ. According to the cell origin of the infiltrate, PCLs can be classified in cutaneous T-cell lymphomas (CTCL) or cutaneous B-cell lymphomas (CBCL). 

Mycosis fungoides (MF) is the most common lymphoproliferative disorder of the epidermotropic, neoplastic T-cell. The expansion of small- to medium-sized memory T-helper lymphocyte (CD45RO and CD4) into a malignant clone shows an affinity for the skin; the memory T-helper clone is mediated by the interaction with dermal capillary endothelial cells and numerous cytokines released by keratinocytes [1]. In this so-called skin stage, the diagnosis is often delayed for an average of six years because of its similarity with inflammatory or infectious dermatoses [2]. Though these malignant cells have an attraction for the skin, after years of progression, they have the ability to lose their skin homing tendency and travel through lymphatics back to the circulation [1]; in the final stages, even an aggressive large cell variant transformation of the disease can occur. In 1806, Alibert et al. was the first to describe MF, and later, in 1876, Bazin et al. defined the stages of the disease (patches, plaques, and tumors) [3].

Over the past twenty years, scientist concerns have focused on developing classifications and, implicitly, treatment guides as accurately as possible for this vast group of pathologies. Using a multidisciplinary approach, WHO has classified lymphoid neoplasm based on their distinct variants. Due to the complexity of this pathology, the development of immunomarkers and permanent research in this field, the classification was periodically updated with new entities [4,5,6,7]. The last update was published by WHO-EORTC in 2018, and it comes with new histologic entities as a result of multiple molecular studies [8]. For a proper clinical approach of the patient, it is important to recognize these distinct forms of lymphoma based on morphology, immunophenotype, molecular and genetics of the disease [5,9,10].

Parapsoriasis en plaque is a group of inflammatory disorders which come with erythematous, well-demarcated, slightly scaly plaques and patches. They are classified into two main groups: small and large plaque parapsoriasis (LPP). If small plaque parapsoriasis (SPP) is widely known as benign, the main focus is on the distinction of LPP as a “benign inflammatory dermatosis” from an incipient form of CTCL. According to certain authors, LPP may be considered a patch stage of MF [11,12,13]. According to others, the same chronic dermatosis is interpreted as a harmless disease which can evolve into cutaneous lymphoma due to a chronic antigen stimulation [14]. The histological sections of LPP describe superficial lymphocyte infiltration with different degrees of epidermotropism, with more than 30% of the cases being described to develop MF [2]. To this day, the disease known as large plaque parapsoriasis is associated with confusion and imprecise definition, due to unspecific clinical appearance, unpredictable evolution, and difficult histologic diagnosis. 

Mycosis fungoides and large plaque parapsoriasis evolution provided intriguing data and are the cause of numerous debates. There is an imperative need to understand the differences and similarities between large plaque parapsoriasis and mycosis fungoides. We aim to identify and compare the genetic alterations and molecular events pathogenicity of these two diseases, trying to respond to every clinician’s dilemma: “Do I treat this LPP case as a benign pathology or as an early cutaneous lymphoma?”. At the same time, in this area of interest, copy number alterations (CNAs) may play an essential role in the genesis of cancer out of genes expression dysregulation. Studies in this field might potentially clarify lymphomagenesis, discover new diagnostic molecules, or even guide targeted therapeutic approaches.

## 2. Materials and Methods

This study was approved by the ethical committee of “Victor Babeș” University of Medicine and Pharmacy Timișoara, Romania and conducted in accordance with the Declaration of Helsinki. All samples were collected with full donor authorization based, on enough information and adequate understanding of the implication and purpose of the study. Every patient assigned a written consent after receiving a consent form and a participant information document. For the patients under 18 years, their tutor signed the written consent. 

### 2.1. Study Population and Inclusion Criteria

The patients were prospectively selected from the University Clinic of Dermatology and Venereology Timișoara, Romania. We selected the cases that were clinically related to MF and LPP, and separated them into two groups (MF group and LPP group). All cases of mycosis fungoides in our group were in patch stage. For the definition of early MF, we followed the algorithm criteria recommended by the ISCL Task Force and the guide published by Pimpinelli et al. [15,16]. 

For every patient, we took a skin biopsy and cryopreserved it at −80 °C. In some cases, when the appearance of the eruption changed its characteristics in time, multiple biopsies were taken. 

The specimens were then diagnosed, on the basis of histological, immunohistochemical and morphological features, by two independent skin pathology specialists. The cases were considered eligible when they met clinical, histopathologic and immunopathologic criteria for the group that they were part of (MF and LPP). Every inclusion criterion followed closely the international diagnostic guidelines. The patient/sample was excluded from our study if the inclusion criteria were not meet.

Table 1 and Table 2 comprise the distribution of the study by diagnosis, gender, and age of the patients.

### 2.2. Genotyping and Statistical Analysis

From these samples, we extracted DNA using single nucleotide polymorphism (SNP) data. The use of SNP array for copy number profiling is a promising approach for the genome-wide analysis. SNP arrays represent whole genome oligonucleotide arrays, which consist of short DNA segments. Many structural abnormalities are associated with small deletions or duplications, and such abnormalities may be revealed with SNP analysis. 

Genomic DNA was extracted using the PURELink Genomic DNA mini kit. The spectrophotometric analysis indicated that extracted DNA was free from protein contamination. The DNA from fresh frozen material was validated on 1% agarose gel electrophoresis. DNA fragments fell between 20 and 30 kb. Real-time PCR was performed on ABI 7900 in 7 uL total volume, with 1 ng of DNA/reaction. The data were processed in accordance with the GATK best practices. 

The copy number data were then imported into the arrayMap online oncogenomic repository system. Quantitative and qualitative alterations were analyzed and mapped to 1 Mb genomic intervals. The copy number alterations distribution (gain/loss) were then plotted into a heatmap in order to compare those two pathologies (MF vs. LPP) in terms of similarities and differences.

### 2.3. T-Cell Receptor Assessment

The diagnosis of mycosis fungoides (MF) represents a challenge in dermatopathology. The early stages of MF have been shown to be characterized by band-like dermal infiltrate of reactive and neoplastic T lymphocytes; exceedingly difficult to distinguish from benign skin conditions such as parapsoriasis. A positive diagnosis often requires other ancillary techniques, such as molecular studies for identifying the T-cell receptor (TCR) gene rearrangement. We evaluated PCR-based T-cell clonality using genomic DNA, to define a differentiation line between MF and LPP.

### 2.4. Purpose and Impact

Our research focuses on the progression path of inflammatory dermatosis to cutaneous lymphomas. The typical example in dermatology for the progression of an inflammatory disease to a lymphoma is the relation between large plaque parapsoriasis (LPP) and mycosis fungoides (MF). 

The imperative need for more comprehensive and interrelated data for studying the genesis and progression of cutaneous lymphoma (CL) is evident.

Due to the heterogeneity of MF and LPP and the scarcity of the cases, there are a very small number of studies that have identified molecular changes in these pathologies.

We aim to identify and compare DNA copy number alteration and gene expression changes between MF and LPP to highlight the similarities and the differences between these pathologies. We want to identify genes and loci capable of differentiating between MF and LPP. We expect a high level of impact of our research on understanding lymphomagenesis, MF biology, and the approach of so called “benign lymphoproliferation”, such as LPP. The data will be integrated with the results previously published in the scientific literature.

With our work, we focus on recognizing molecular events involved in MF pathogenicity, decipher novel molecular events linked to the biology of this disease, and highlight genetic alterations in MF vs. LPP, leading to the develop of new accurate diagnostic tools.

Molecular studies are indispensable to better explore the pathogenesis and clonal evolution of lymphoproliferative skin diseases. A better understanding of the cell biology, immunology and genetics underlying the development and progression of cutaneous lymphoma will lead to the design of more rational treatment strategies.

This may have an immediate impact on the prognosis and, in the future, an impact on the implementation of targeted and personalized therapies using candidate molecules.

At the same time, the complex data provided by this study will add important information regarding the understanding and classification of, and the differences between, these two diseases.

## 3. Results

The morphology and histology of MF can resemble LPP and vice versa. These similarities make it difficult to interpret and differentiate the characteristics between them. Although, as mentioned before, LPP is considered a typical presentation of MF by some authors, it is obvious that the real essence of LPP physiopathology is still questionable, along with its diagnosis and treatment. Over the last 10 years, new molecular techniques began solving the big and complicated puzzle of the CL spectrum disease. They identified genetic alterations and genes expression that are able to differentiate a malignant disease from a benign one, and also genes that can influence the prognosis and evolution of the pathology. 

To date, several genetic abnormalities have been highlighted in MF, with gains being described more often than deletions in relation to copy number alterations (CNAs) [10]. Gains were emphasized to affect chromosomes 1, 7, 8, 9qter, 17, 19, 22 and losses on the chromosomes 6q, 9pter, 10, 13q, 16q and 17p [10].

### 3.1. CNAs in MF vs. LPP

**Figure 1:**
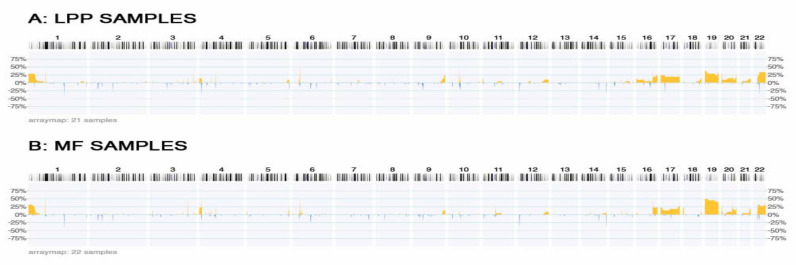
Frequency of genomic gains and losses in LPP (A) and MF (B). The heat map shows the frequency of gains (orange, up)/losses (blue, down), ordered from chromosome 1–22. For example, a frequency of 25% means that, in 25% of the selected cases, a variation in the copy numbers (deletions or duplications) was observed.

We have analyzed the genomic abnormalities found in 21 samples of LPP and 22 samples of MF and summarized them in Figure 1. The study retrieval is found on Table 1 and the patients’ characteristics are highlighted in Table 2.

After reviewing each group (Figure 1), we observed that the histograms generated for chromosomes 1–22 (Figure 1), were remarkably similar, and they had a lot of CNAs in common. As previously described, duplications are more common than deletions, for MF and, in our case, LPP. Duplications are seen on chromosome 1p, some loci on chromosomes 3 and 4p, a spike on 6p and 9qter, and some spikes on chromosome 10, 12qter, 18qter, and 21. However, the most affected are chromosomes 16, 17, 19, 20 and 22. Deletions are rarely seen as small portions of the DNA on chromosomes 1, 2, 4, 6, 12, 14 and 22. 

### 3.2. CNAs on Chromosome 1 in LPP vs. MF

For chromosome 1, we observed on both MF and LPP important gains on the short arm with a duplication spike on 1p31.1, which comprised almost 50% of the cases in both groups. An important difference between the histograms is seen on the interval 1p33-p31.1, where we have several duplications in LPP that are missing in MF. To initiate the theory that this loci interval can be a “protective” CNA or a benign sign, further research needs to be done, maybe with larger number of cases. 

These duplications correspond to various genes’ loci. The gain on 1p36.22 corresponds to the mTOR gene, which promotes proliferation of the cell, acceleration of its metabolism, contributes to tumor progression and downregulates autophagy [17]. The same locus belongs to the PIK3CD gene, which is strongly related to mTOR. The activation of these genes in cancer made the scientific family focus on targeting and down-streaming them, with the hope that all hallmark of pathways that come with these duplications could be stopped, along with the disease [18].

In terms of deletions on this chromosome, the most important losses in our study groups are seen next to 1q21.3.

### 3.3. CNAs on Chromosome 2 in LPP vs. MF

Chromosome 2 has three deletion spikes highlighted in Figure 1. They correspond to loci 2p22, 2p16 and 2q11. 

ZAP70 gene (2q11.2) belongs to the PTK family and it plays an important role in Th lymphocyte activation and T-cell development. Mutations of this gene are causing T-cell defects and are incriminated in chronic lymphocytic leukemia [19,20].

### 3.4. CNAs on Chromosome 3 in LPP vs. MF

On chromosome 3, seen in Figure 1, near 3p21.3, there is a deletion on the MF histogram that we cannot find on the LPP histogram. Tumor suppressor genes, which have been described and isolated in this chromosome region, play a critical role in tumorigenesis [21]. These genes are BAP1, CACNA2D2, DLCI, FUS1, H37, HYAL1, RASS1A, SEMA3B and SEMA3F [21].

Another difference between the LPP group and the MF group is found on p12.3 loci, where a few copy numbers are lost in LPP but not in MF.

At the same time, we observe gains on 25% of the LPP cases on 3q27, and an important duplication on both LPP and MF samples near 3q25.3. In the MF group, gains in copy number near q25 reach almost half of the cases, and 25% of cases in the LPP group. This locus is known to be occupied by the MLF1 gene. Myeloid leukemia factor 1 (MLF1) is an oncoprotein with a role in the phenotypic determination of primary hematopoietic progenitor cells [22]. Aberrant genetic alterations of this gene increase the predisposition to oncogenesis due to its role in the stabilization and negative regulator of p53 activity [23]. Copy number losses of this gene were also observed and studied by Mansur M. et al. in infants with T-cell acute lymphoblastic leukemia [24].

### 3.5. CNAs on Chromosome 4 in LPP vs. MF

We can also notice a lot of similarities on chromosome 4, such as gains on the short arm of this chromosome. One important difference is highlighted on 4p16.1, where we can find a gain spike on the MF group and a loss spike on the LPP group. 

In humans, on 4p16, the S100P gene is mapped; a relatively “young” gene with a range of various functions, from cellular behavior to the development of cancer [25].

### 3.6. CNAs on Chromosome 5 in LPP vs. MF

This chromosome highlights very few CNAs both on MF and LPP, but we can see a difference in the number of gains on 5q35.3, which covers 25% of the MF cases. At the same time, this locus is covered by important losses in the LPP group.

### 3.7. CNAs on Chromosome 6 in LPP vs. MF

Reviewing chromosome 6, gains on 6p22.1–21.3, with a spike on p21.32-p21.33, are seen in both MF and LPP. Another duplication is retrieved on the long arm of the chromosome, on q27 loci, only on the LPP histogram. In terms of losses, q14.1 is noteworthy, with a few cases both on the LPP and the MF group. On the other hand, deletion of q16.3 is seen only on LPP cases. 

Tumor necrosis factor (TNF) and its cancer promoting capacity, out of its role in proliferation, differentiation, and apoptosis, drew the experts’ attention. Its genetic localization is on the short (p) arm of chromosome 6, at position 21.33. Cancer biology and its proliferation, migration, angiogenesis, survival, and invasion it is promoted by TNF [26]. 

### 3.8. CNAs on Chromosome 9 in LPP vs. MF

The genomic regions of chromosome 9, with frequent imbalances, comprise the long arm (q) at position 34. These imbalances are represented by copy number gains on MF, and more predominantly on LPP (Figure 1).

The NOTCH1 gene, whose cytogenetic location is on 9q34.3, has been considered both a tumor suppressor gene and an oncogene. As an oncogene, it has the potential role of transforming normal cells into malignant ones, promoting survival and uncontrolled proliferation. However, as a tumor suppressor, it prevents the development of cancer through apoptosis. Researchers are still trying to understand why both activating and inactivating this gene can lead to cancer development [27].

The cytogenetic region of the CDKN2A gene (9p21.3) was observed to be deleted in one case of MF. CDKN2A provides instructions for important tumor suppressor genes such as p16 and p14 [28].

### 3.9. CNAs on Chromosome 10 in LPP vs. MF

Downregulations of the FAS gene (10q23.31), which modulates the caspase cascade and tumor suppressor PTEN (10q23.31), are described in numerous CTCLs [10,29,30]. In our samples, near this cytogenetic location, deletions are affecting very few cases on both MF and LPP samples.

Regarding duplications, near q11.22, copy number gains can be seen in both groups, but more significant in LPP samples. The LPP group is affected on the same locus by important deletions.

### 3.10. CNAs on Chromosome 11 in LPP vs. MF

In Figure 1, chromosome 11, we spot two cytogenetic locations that are affected by gains. On the LPP histogram, we can easily point out the p15.5 DNA locus duplicated in almost 25% of the cases. On the other heat map, which belongs to MF samples, in a quarter of the cases gains affect location near the centromere.

### 3.11. CNAs on Chromosome 12 in LPP vs. MF

Deletions on p13 are seen on chromosome 12 in both groups, almost in the same proportions but with a little higher incidence in MF samples. This locus is harboring the CDKN1B gene, which provides instructions for p27 protein. P27 plays an essential role in controlling division and cell growth by blocking the cell cycle. As a result, somatic mutations in the CDKN1B gene are associated with cancer development, aggressive tumors, and poor prognosis [31].

### 3.12. CNAs on Chromosome 14 in LPP vs. MF

Regarding chromosome 14, what attracts attention are the two deletion spikes encountered in both heat maps (Figure 1). The first one is located on 14q24.2-q24.3, which belongs to the NUMB gene, and second one is seen on the qter molecular region, near the XRCC3 gene location.

NUMB acts in a similar way to a tumor suppressor gene, by regulating Notch pathways. This downregulation allows tumors to evolve. This triggering cancer phenotype was described in numerous cancers such as breast cancer, salivary gland carcinoma, non-small-cell lung carcinoma, malignant pleural mesothelioma or medulloblastoma [32]. Notch pathway stimulation is also involved in various cancers such as melanomas, leukemias and lymphomas [32].

XRCC3 is a gene with role in repairing DNA and maintaining chromosome stability, found on the log arm (q) of chromosome 14, at position 32.33 [33]. This gene downregulation is associated with neoplasia in patients with radiosensitivity and is incriminated in numerous cancers such as malignant melanoma, gastric cancer, lung, gynecological and breast tumors [33,34]. 

### 3.13. CNAs on Chromosome 16 in LPP vs. MF

In Figure 1, recurring gains could be observed on chromosome 16. LPP duplications comprise the entire surface of the chromosome but are more predominant on the terminal part of the q arm. In the MF histogram, gains are starting from 16q22.1 to the end of the chromosome. A duplication spike is seen near band 16q22.1 on both groups, but is predominant on MF samples, which comprise 50% of the cases.

### 3.14. CNAs on Chromosome 17 in LPP vs. MF

The genome abnormalities include almost exclusively duplications on the entire surface of chromosome 17, affecting the MF and the LPP group almost in the same proportion. It is well-known that this chromosome hosts a large group of genes implicated in oncogenesis, such as the STAT gene, the SOCS3 gene that regulates JAK/STAT pathway, the RARA gene and the BIRC5 gene. In his study, Karenko et al. links aberration of chromosome 17 with active or progressive CTCL [35].

What differentiates the two histograms represented for chromosome 17 are the two deletions. LPP has a deletion near 17p11.2 in almost 25% of the cases, and MF samples present losses on 17q21.31.

Deletions on the short arm (p) and the deletion of the p53 gene described in other studies are seen in neither of our MF or LPP samples [10]. This could have happened because of the relatively small group of patients. 

### 3.15. CNAs on Chromosome 18 in LPP vs. MF

When it comes to chromosome 18, we spot an important difference which consists of gains in p11.32, retrieved only on MF samples and with an incidence of 25%. 

This cytogenetic location belongs to genes such as NDC80, known to be highly expressed in cancer [36].

At the same time, we noted duplications on q21.31–q23 on the LPP histogram, with only q23 gains being traced in MF samples, in addition.

### 3.16. CNAs on Chromosome 19 in LPP vs. MF

Chromosome 19 is the most affected by CNAs in our study, both on LPP and MF. Gains are seen in the MF group in 50% of the samples on the entire surface of the chromosome. These recurring duplications, affecting chromosome 19 in MF, have been previously described by scientists [10,37]. Important genes that impact cancer, such as CD70, JAK3 and GDF15, are found on ch19.

The LPP histogram reveals gains on the whole chromosome, but in a lower proportion than MF (Figure 1).

### 3.17. CNAs on Chromosome 20 in LPP vs. MF

As previously described, gains also affect chromosome 20 almost entirely, in both sample groups. Three peaks can be easily observed on both MF and LPP, near p13, q13.12 and q13.33. 

### 3.18. CNAs on Chromosome 21 in LPP vs. MF

Duplications of chromosome 21 start from the same location (p11.1), affecting a bigger proportion the long (q) arm, near region 22.3. These CNAs affect both MF and LPP equally.

### 3.19. CNAs on Chromosome 22 in LPP vs. MF

Near the centromere of chromosome 22, gains are starting to affect both pathologies. The short arm is free of CNAs in MF and LPP. The heat map (Figure 1) distributes the duplications a little bit differently. Large plaque parapsoriasis has a larger number of gains on the q11.23–q13.2 region. The mycosis fungoides histogram reveals a larger number of CNAs near q11.21–q11.22 and q13.31–q13.33. 

Regarding copy number losses on this chromosome, what attracts our attention are two regions: q11.21, which is more predominant in the MF group, and q11.23, which is more consistent in the LPP samples.

CNA differences between MF and LPP are summarized in Table 3. In LPP, copy number gains were seen in cytogenetic locations occupied by genes such as JUN, CDKN2C, BCL6, HRAS, CD151, FUS, SOCS1, MALT1, BCL2, BCR, XRCC, and FGFR10P. Losses of genetic material seen in our LPP group affected loci known to be occupied by ROBO1, FGFR3, NSD1, FLT, HACE1 and FLCN genes. On the other hand, our MF group was affected by gains on S100P, NSD2, NSD1, FLT4, COL1A1, NDC80, CTCL1, MAPK1, MLC1 DNA location genes. MF CNA losses highlighted genes such as BAP1, FUS1, and COL1A1. All these CNAs were seen in a frequency between 5% and 40% of the samples (Figure 1, blue-down-losses, orange-up-gains).

CNA similitudes between the MF group and the LPP group are grouped in Table 4. This table comprise CNAs that affect both lots, but one of those two pathologies has a higher frequency of affected samples. Deletions of DNA material affected locations near MLLT111 (next to BCl9), BCL11A, CDKN1B, CD27, HEBP1, XRCC3, and CTCL1 in MF and ZAP70, CXCL9, IL15, and BCR in LPP (blue, down, Figure 1). Gains (orange, up, Figure 1) are highlighted in the proximity of FUB1 (next to JAK1), mTOR, PIK3CD, MLF1, IL12A, RHOH, FGFR3, KIT, CD40 in the MF group. NOTCH1 and SOX18 are duplicated in the LPP group. Duplications on chromosome 19 are more consistent in the MF group. The CNA frequency of the affected samples was between 25% and 50%. We observed interesting CNA locations such as 6p21.32–33, 6q14.1, 14q24.2–24.3, 20p13 and chromosome 17, which affects both groups equally. At the same time, in the LPP group, 10q11.22 is affected both by deletions and duplications.

### 3.20. T-Cell Receptor Rearrangements

TCR clonality assay was carried out for both groups, to provide evidence in order to facilitate the diagnosis of these two pathologies. As seen in Table 5, TCR rearrangements affected 13 samples from the MF group and five from the LPP group. We can observe an important difference between these very similar pathologies; TCR clonality tends to be more present in MF samples than in LPP. As described in the literature, malignant αβ TCRs are expressed in MF. In addition, large plaque parapsoriasis with TCR rearrangements can predict a neoplastic transformation, which may correspond to an early MF. However, precaution must be taken and TCR follow-up of non-clonal cases is highly required, especially when the clinical aspect of the eruption changes or symptomatology, such as pruritus, appears.

## 4. Discussion

Medicine is not mathematics all in black and white; not all cases follow the same patterns, so we have grey shades. When diagnosing MF, we must follow numerous criteria and take into account all clinical, morphological, histological, immunohistochemical and genetical data. All of this often requires a follow-up of the case over a longer period of time, especially in those who are borderline or premalignant. On one hand, so as not to miss a diagnosis that requires special care, and on the other hand to avoid the aggressive treatment of a benign case with therapies that sometimes have significant adverse effects. 

Regarding copy number alterations, our study was in accordance with other publications and highlighted that gains were more consistent than deletions in both MF and LPP, affecting chromosomes 16, 17, 19, 20 and 22. The high frequency of duplications on chromosome 7 and 8, described in other studies, was not seen in our groups [10]. The relatively low number of cases, due to the small incidence of these pathologies, resulted in what we consider to be the limitation of this study, and can maybe explain the absence of CNAs in chromosome 7 and 8. 

In our study groups, chromosome 1 showed a hallmark of common alterations between LPP and MF, such as duplications near mTOR, PIK3CD and other protooncogenes. In a five-year follow-up of CTCL patients, Karenko et al. concluded that aberrations of chromosome 1 are a hallmark of an existing cutaneous lymphoma, even if in remission [35].

Based on our observation, other CNAs common for both MF and LPP are deletions on chromosome 2 near the ZAP70 gene, gains on 3q near the MLF1 gene, and gains on 4p. On the short arm of chromosome 6 we found gains on 22.1–21.3 cytogenetic locations, near the TNF gene. Higher TNF gene expression levels increase one’s risk of developing various types of cancer, including non-Hodgkin’s lymphoma [26]. In other studies, MF losses on chromosome 6 were more predominant that in our lot [10].

Recurring and important duplications affect chromosomes 16, 17, 19, 20, 21 and 22, on large portions of DNA, as previously described in the literature [10]. MF cases seem to be more frequently affected by these gains than LPP cases. 

Other CNA differences between LPP and MF spotted in our study, are revealed in Table 3. In Table 3, 4p16.1 and q35.3-qter draw our attention because of the opposite way of affecting DNA in MF vs. LPP. These loci can be major distinguishing factors between benign and malign, being described in the literature as one of the most recurrent gains in MF and SS [38].

These cytogenetic locations require more research to see whether they have an impact on future molecular diagnosis differentiation between mycosis fungoides and large plaque parapsoriasis.

TCR sequencing provides significant information about early-stage malignancy lesions and it is a strong predictor of disease progression and poor survival in MF patients. At the same time, it is a promising marker for the identification of malignant T-cell proliferation. This study found a greater incidence of TCR gene rearrangements in MF, comprising 45% (23% in LPP cases). TCR rearrangement in LPP can predict the evolution to MF spectrum disease. Due to various common clinical, morphological, and molecular events, negative TCR cases require close attention and a mandatory follow-up, given that 20% of LPP will transform into CTCL [39]. According to Zaaroura et al., clonal T-cell receptor gene rearrangements may serve as clues of parapsoriasis evolving to MF [40].

## 5. Conclusions

This study has taken a step forward in finding out the differences and similarities between MF and LPP, for a more specific and implicitly correct approach of the case. Recently, DNA profiling has become an extremely useful tool for the diagnosis and therapy of important pathologies on the oncological spectrum. As noted above, in our study results, the similarity between the two pathologies in terms of CNAs is striking, emphasizing once again the difficulty of approaching and differentiating them. Modern medicine must assume that there is often a very fine line between a benign and a malignant pathology. Understanding and unravelling the transition between benign and early phase malignant requires experience, a close and timely follow-up, and an understanding of the pathomechanisms of malignancy.

## Figures and Tables

**Table 1:** Study retrieval.

Group	No. of Samples	No. of Patients	Gender M/F	Mean Age
**LPP**	21	21	13/8	65
**MF**	22	18	11/7	63

Abbreviation: LPP—Large plaque Parapsoriasis, MF—Mycosis fungoides, M/F—Masculine/Feminine.

**Table 2:** Characteristics of the patients enrolled in the study.

Patient No.	Diagnosis LPP/MF	Gender M/F	Age (Years)
**1**	LPP	F	66
**2**	LPP	M	61
**3**	LPP	M	58
**4**	LPP	M	25
**5**	LPP	F	71
**6**	LPP	F	60
**7**	LPP	F	69
**8**	LPP	F	67
**9**	LPP	M	59
**10**	LPP	M	59
**11**	LPP	M	64
**12**	LPP	M	78
**13**	LPP	M	59
**14**	LPP	F	80
**15**	LPP	M	67
**16**	LPP	F	67
**17**	LPP	M	63
**18**	LPP	F	63
**19**	LPP	M	75
**20**	LPP	M	72
**21**	LPP	M	80
**22**	MF	M	63
**23**	MF	M	63
**24**	MF	M	77
**25**	MF	F	78
**26**	MF	F	70
**27**	MF	M	59
**28**	MF	F	71
**29**	MF	F	72
**30**	MF	M	80
**31**	MF	M	64
**32**	MF	F	78
**33**	MF	M	79
**34**	MF	M	14
**35**	MF	F	29
**36**	MF	M	61
**37**	MF	F	56
**38**	MF	M	40
**39**	MF	M	71

**Table 3:** Summary of CNA differences between MF and LPP.

CHR. NO.	CYTOGENETIC LOCATION	CNA GAINS/LOSSES	PROXIMITY GENES	CNA FREQUENCY	MF/LPP
**1**	p33–p31.1	G	TAL, STIL, EPS15, JUN, CDKN2C	5%	LPP
**3**	p21.3	L	BAP1, CACNA2D2, DLCI, FUS1, H37, HYAL1, RASS1A, SEMA3B and SEMA3F	5%	MF
**3**	p12.3	L	ROBO1	20%	LPP
**3**	q27	G	BCL6, EIF4A2, LPP, locus near TP63-3q28	25%	LPP
**4**	p16.1	G	S100P (4p16.1) TACC3, NSD2, FGFR3 and CTBP1 (all on 4p16.3),	40%	MF
**4**	p16.1	L		30%	LPP
**5**	q35.3–qter	G	NSD1, FLT4	25%	MF
**5**	q35.3–qter	L		25%	LPP
**6**	q16.3	L	HACE1, LIN28B	20%	LPP
**6**	q27	G	CCR6, PDCD2, FGFR10P	20%	LPP
**11**	p15.5	G	HRAS, CD151, CD81, IGF2	25%	LPP
**16**	p arm	G	FUS, ITGAM, IL32, SOCS1	10%	LPP
**17**	p11.2	L	FLCN, SHMT1	25%	LPP
**17**	q21.31	L	COL1A1, TBX21, HOXB, DLX4	15%	MF
**18**	p11.32	G	NDC80, TYMS, ADCYAP1, YES1	25%	MF
**18**	q21.31–q23	G	MALT1, BCL2	10%	LPP
**22**	q11.23–q13.2	G	BCR, MMP11, SOX10, XRCC6, MLK1	30%	LPP
**22**	q11.21–q11.22	G	CRKL, CTCL1, SEPT5, MAPK1, PRAME	30%	MF
**22**	q13.31–q13.33	G	MLC1	30%	MF

**Table 4:** Summary of common CNAs that affect predominantly LPP or MF.

CHR. NR.	CYTOGENETIC LOCATION	CNA GAINS/LOSSES	PROXIMITY GENES	CNA FREQUENCY	MF/LPP
**1**	1p31.1	G	FUBP1 gene (next to JAK1 gene 1p31.3)	50%	MF
**1**	1p36.22	G	mTOR, PiK3CD	25%	MF
**1**	1q21.3	L	MLLT111 (next to BCL9 gene 1q21.2)	40%	MF
**2**	2p22	L	next to DNMT3A 2p23	25%	MF
**2**	2p16	L	BCL11A, FBXO11	25%	MF
**2**	2q11	L	ZAP70, AFF3	25%	LPP
**3**	q25	G	MLF1, GMPS, IL12A	50%	MF
**4**	p	G	PDGFRA, RHOH, FGFR3, KIT, IGFBP7, CXCL9, EIF4E, LEF1, IL15, ING2	25%	MF
**4**	p	L		25%	LPP
**6**	p21.32–21.33	G	NOTCH4, TAP1, DAXX, HLA genes, TAP2, PU5F1, TNF, LTB, LTA	50%	both
**6**	q14.1	L	PHIP	25%	both
**9**	q34	G	NOTCH1, ABL1, NUP214, FPGS,	25%	LPP
**10**	q11.22	G and L	MAPK8, NCOA4, MSMB, ANXA8, RBP3	30%	LPP
**12**	p13	L	CDKN1B, KDM5A, M6PR, HEBP1, AICDA, CD27, CD9, FGF23	35%	MF
**14**	q24.2–24.3	L	NUMB	25%	both
**14**	q32.33	L	XRCC3	40%	MF
**16**	q22.1	G	NQO1, CBFB, HAS3	50%	MF
**17**	entire chromosome	G	STAT, SOCS3, RARA, BIRC5, CD79B, CD68, CCR7, PRKCA, MLLT6	25%	both
**19**	entire chromosome	G	CD70, JAK3, JUND, ICAM1, GNA11, CD79A, GDF15, DNMT1, LDLR, ACTN4, IRF3, CD33	50%	MF
**20**	p13	G	CDC25B	30%	both
**20**	q13.12	G	CD40	30%	MF
**20**	q13.33	G	SOX18, GATA5, TNFRSF6B	25%	LPP
**22**	q11.21	G	CRKL, CTCL1, SEPT5	20%	MF
**22**	q11.23	G	BCR, MMP11	30%	LPP

**Table 5:** TCR assessment by PCR.

Pathology	TCR α	TCR β
**Mycosis Fungoides**	3	10
**Large Plaque Parapsoriasis**	-	5

## References

[1] http://emedicine.medscape.com/article/2139720-overview#a4.

[2] Fujii K., Kanekura T. (2019). Next-Generation sequencing technologies for early-stage cutaneous T-Cell Lymphoma. Front. Med..

[3] Zackheim H.S. (2004). Cutaneous T-Cell Lymphoma: Mycosis Fungoides and Sezary Syndrome.

[4] Campo E., Swerdlow S.H., Harris N.L., Pileri S., Stein H., Jaffe E.S. (2011). The 2008 WHO classification of lymphoid neoplasms and beyond: Evolving concepts and practical applications. Blood.

[5] Swerdlow S.H. (2008). ECNH. Classification of Tumours of Haematopoietic and Lymphoid Tissues.

[6] (2001). WHO Classification of Tumors: Pathology and Genetics of Tumors of Hematopoietic and Lymphoid Tissues.

[7] Willemze R., Kerl H., Sterry W., Berti E., Cerroni L., Chimenti S., Diaz-Peréz J.L., Geerts M.L., Goos M., Knobler R. (1997). EORTC classification for primary cutaneous lymphomas: A proposal from the Cutaneous Lymphoma Study Group of the European Organization for Research and Treatment of Cancer. Blood.

[8] Willemze R., Cerroni L., Kempf W., Berti E., Facchetti F., Swerdlow S.H., Jaffe E.S. (2019). The 2018 update of the WHO-EORTC classification for primary cutaneous lymphomas. Blood.

[9] Swerdlow S.H., Campo E., Pileri S.A., Harris N.L., Stein H., Siebert R., Advani R., Ghielmini M., Salles G.A., Zelenetz A.D. (2016). The 2016 revision of the World Health Organization classification of lymphoid neoplasms. Blood.

[10] Gug G., Huang Q., Chiticariu E., Solovan C., Baudis M. (2019). DNA copy number imbalances in primary cutaneous lymphomas. J. Eur. Acad. Dermatol. Venereol..

[11] Sanchez J.L., Ackerman A.B. (1979). The patch stage of mycosis fungoides. Criteria for histologic diagnosis. Am. J. Dermatopathol..

[12] Weedon D. (2010). Weedon’s Skin Pathology.

[13] Cerroni L. (2014). Skin Lymphoma: The Illustrated Guide.

[14] Harvey N.T., Spagnolo D.V., Wood B.A. (2015). Could it be mycosis fungoides?: An approach to diagnosing patch stage mycosis fungoides. J. Hematopathol..

[15] Olsen E., Vonderheid E., Pimpinelli N., Willemze R., Kim Y., Knobler R., Zackheim H., Duvic M., Estrach T., Lamberg S. (2007). Revisions to the staging and classification of mycosis fungoides and Sézary syndrome: A proposal of the International Society for Cutaneous Lymphomas (ISCL) and the cutaneous lymphoma task force of the European Organization of Research and Treatment of Cancer (EORTC). Blood.

[16] Pimpinelli N., Olsen E.A., Santucci M., Vonderheid E., Haeffner A.C., Stevens S., Burg G., Cerroni L., Dreno B., Glusac E. (2005). Defining early mycosis fungoides. J. Am. Acad. Dermatol..

[17] Tian T., Li X., Zhang J. (2019). mTOR signaling in cancer and mTOR Inhibitors in solid tumor targeting Therapy. Int. J. Mol. Sci..

[18] David A. (2014). Fruman and christian Rommel. PI3K and cancer: Lessons, challenges and opportunities. Nat. Rev. Drug Discov..

[19] https://ghr.nlm.nih.gov/gene/ZAP70#conditions.

[20] Koczkodaj D., Popek-Marciniec S., Zmorzyński S., Wąsik-Szczepanek E. (2019). Filip AA2. Examination of clonal evolution in chronic lymphocytic leukemia. Med. Oncol..

[21] Xiong W., Zeng Z.Y., Xia J.H., Xia K., Shen S.R., Li X.L., Hu D.X., Tan C., Xiang J.J., Zhou J. (2004). A Susceptibility Locus at Chromosome 3p21 Linked to familial. Cancer Res..

[22] https://ghr.nlm.nih.gov/gene/MLF1#location.

[23] Yoneda-Kato N., Kato J.-Y. (2007). Shuttling imbalance of MLF1 results in p53 instability and increases susceptibility to oncogenic transformation. Mol. Cell. Biol..

[24] Mansur M.B., Delft F.W., Colman S.M., Furness C.L., Gibson J., Emerenciano M., Kempski H., Clappier E., Cave H., Soulier J. (2015). Distinctive genotypes in infants with T-cell acute lymphoblastic leukaemia. Br. J. Haematol..

[25] Prica F., Radon T., Cheng Y., Crnogorac-Jurcevic T. (2016). The life and works of S100P—From conception to cancer. Am. J. Cancer Res..

[26] Wang X., Lin Y. (2008). Tumor necrosis factor and cancer, buddies or foes?. Acta Pharmacol. Sin..

[27] https://ghr.nlm.nih.gov/gene/NOTCH1#conditions.

[28] https://ghr.nlm.nih.gov/gene/CDKN2A#conditions.

[29] Wu J., Siddiqui J., Nihal M., Vonderheid E.C., Wood G.S. (2011). Structural alterations of the FAS gene in cutaneous T-cell lymphoma (CTCL). Arch. Biochem. Biophys..

[30] Cristofoletti C., Picchio M.C., Lazzeri C., Tocco V., Pagani E., Bresin A., Mancini B., Passarelli F., Facchiano A., Scala E. (2013). Comprehensive analysis of PTEN status in Sézary syndrome. Blood.

[31] https://ghr.nlm.nih.gov/gene/CDKN1B#conditions.

[32] Garcia-Heredia J.M., Carnero A. (2018). NUMB and NUMBL differences in gene regulation. Oncotarget.

[33] https://ghr.nlm.nih.gov/gene/XRCC3#location.

[34] http://www.cancerindex.org/geneweb/XRCC3.htm.

[35] Karenko L., Sarna S., Kähkönen M., Ranki A. (2003). Chromosomal abnormalities in relation to clinical disease in patients with cutaneous T-cell lymphoma: A 5-year follow-up study. Br. J. Dermatol..

[36] https://www.genecards.org/cgi-bin/carddisp.pl?gene=NDC80.

[37] Prochazkova M., Chevret E., Mainhaguiet G., Sobotka J., Vergier B., Belaud-Rotureau M.A., Beylot-Barry M., Merlio J.P. (2007). Common chromosomal abnormalities in mycosis fungoides transformation. Genes Chromosomes Cancer.

[38] Vermeer M.H., van Doorn R., Dijkman R., Mao X., Whittaker S., van Voorst Vader P.C., Gerritsen M.J.P., Geerts M.L., Gellrich S., Söderberg O. (2008). Novel and highly recurrent chromosomal alterations in Sézary syndrome. Cancer Res..

[39] Bernier C., Nguyen J.M., Quéreux G., Renault J.J., Bureau B., Dreno B. (2007). CD13 and TCR Clone: Markers of Early Mycosis Fungoides. Acta Derm. Venereol..

[40] Zaaroura H., Sahar D., Bick T., Bergman R. (2018). Relationship between pityriasis lichenoides and mycosis fungoides: A clinicopathological, immunohistochemical, and molecular study. Am. J. Dermatopathol..

